# Finger Posture and Finger Load are Perceived Independently

**DOI:** 10.1038/s41598-019-51131-x

**Published:** 2019-10-21

**Authors:** Brendan Prendergast, Jack Brooks, James M. Goodman, Maria Boyarinova, Jeremy E. Winberry, Sliman J. Bensmaia

**Affiliations:** 1 Department of Organismal Biology and Anatomy, University of Chicago, Chicago, IL USA; 2 Committee on Computational Neuroscience, University of Chicago, Chicago, IL USA

**Keywords:** Sensory processing, Somatosensory system

## Abstract

The ability to track the time-varying postures of our hands and the forces they exert plays a key role in our ability to dexterously interact with objects. However, how precisely and accurately we sense hand kinematics and kinetics has not been completely characterized. Furthermore, the dominant source of information about hand postures stems from muscle spindles, whose responses can also signal isometric force and are modulated by fusimotor input. As such, one might expect that changing the state of the muscles – for example, by applying a load – would influence perceived finger posture. To address these questions, we measure the acuity of human hand proprioception, investigate the interplay between kinematic and kinetic signals, and determine the extent to which actively and passively achieved postures are perceived differently. We find that angle and torque perception are highly precise; that loads imposed on the finger do not affect perceived joint angle; that joint angle does not affect perceived load; and that hand postures are perceived similarly whether they are achieved actively or passively. The independence of finger posture and load perception contrasts with their interdependence in the upper arm, likely reflecting the special functional importance of the hand.

## Introduction

Proprioception – the sense of our body’s configuration, movement, and of the forces we exert – is critical for the planning and execution of movement as evidenced by the crippling deficits that result from its loss^1–3^. Much of the work investigating proprioception in the upper limb has focused on proprioception of its proximal aspect — the arm^4–6^. However, the arm and hand serve two distinct functional roles, with the former tasked with transporting the hand in three-dimensional space and the latter tasked with conforming to and manipulating objects of various shapes and sizes. Indeed, cortical representations of the arm and hand are specialized in a manner that reflects their different functions: The representation of the hand in primate somatosensory cortex is magnified with respect to that of the proximal limb^7^; and the primary motor cortex (M1) of primates contains a distinct caudal module specialized for direct control of the hand^8^. As such, one may also anticipate differences to emerge between the arm and hand in terms of the perceptually accessible proprioceptive information.

In particular, the perceived configuration of our arms can be distorted by a variety of factors. For example, perceived arm position is biased by loads supported by the arm^9,10^ and vice versa^5^, by muscle contraction history^11^, and depends on whether posture is achieved actively or imposed on the arm^12^. In other words, signals related to arm kinematics and kinetics cannot be entirely perceptually disentangled.

Muscle spindle afferents, which are thought to be the main sources of proprioceptive signals^3,11^ individually convey ambiguous information about limb position: In addition to muscle length, muscle tension and history^13^ and fusimotor input^14^ also influence spindle responses, which likely underlies the influence of imposed torque and motor intent on perceived arm posture. The question remains whether the ambiguity between postural and load-related signals is also observed for hand proprioception.

To address this question, we performed a series of experiments in which we assess the degree to which finger joint position is affected by a load applied to that joint and whether perceived load is affected by joint position. We also examined whether perceived posture depends on whether the posture was achieved actively or whether it is imposed by the experimenter. We compared active and passive movements to test the possibility that acuity is influenced by the different fusimotor states imposed by these two conditions. We found that neither load nor intent (active vs. passive) substantially affects perceived position and that finger joint position does not affect perceived load. We infer that the function of the hand requires a precise, unambiguous, perceptually-accessible readout of position and load and that this readout is supported by specialized processing of hand proprioception downstream of the proprioceptive afferents themselves.

## Methods

### Subjects

A total of 42 subjects participated (24 male, 18 female; mean age: 22.2 (16–32); 39 right-handed, 1 left-handed, 2 with no declared preference). Two subjects participated in two protocols. Specifically, eight subjects participated in active joint angle matching, eight in passive joint angle matching, eight in angle discrimination, six in the first weight discrimination experiment using consistent finger angles (30 degrees), eight in the second weight discrimination experiment using different finger angles (10 and 60 degrees), and six in the third weight discrimination experiment testing whether cutaneous cues were sufficient. All subjects reported normal tactile sensitivity and no neurological deficits. All methods were carried out in accordance with relevant guidelines and regulations. Experimental procedures were approved by the Institutional Review Board of the University of Chicago. Informed consent was obtained from each subject, and for subjects younger than 18 years, informed consent from a parent or legal guardian was also obtained. Subjects were compensated for their participation.

### Apparatus

**Figure 1:**
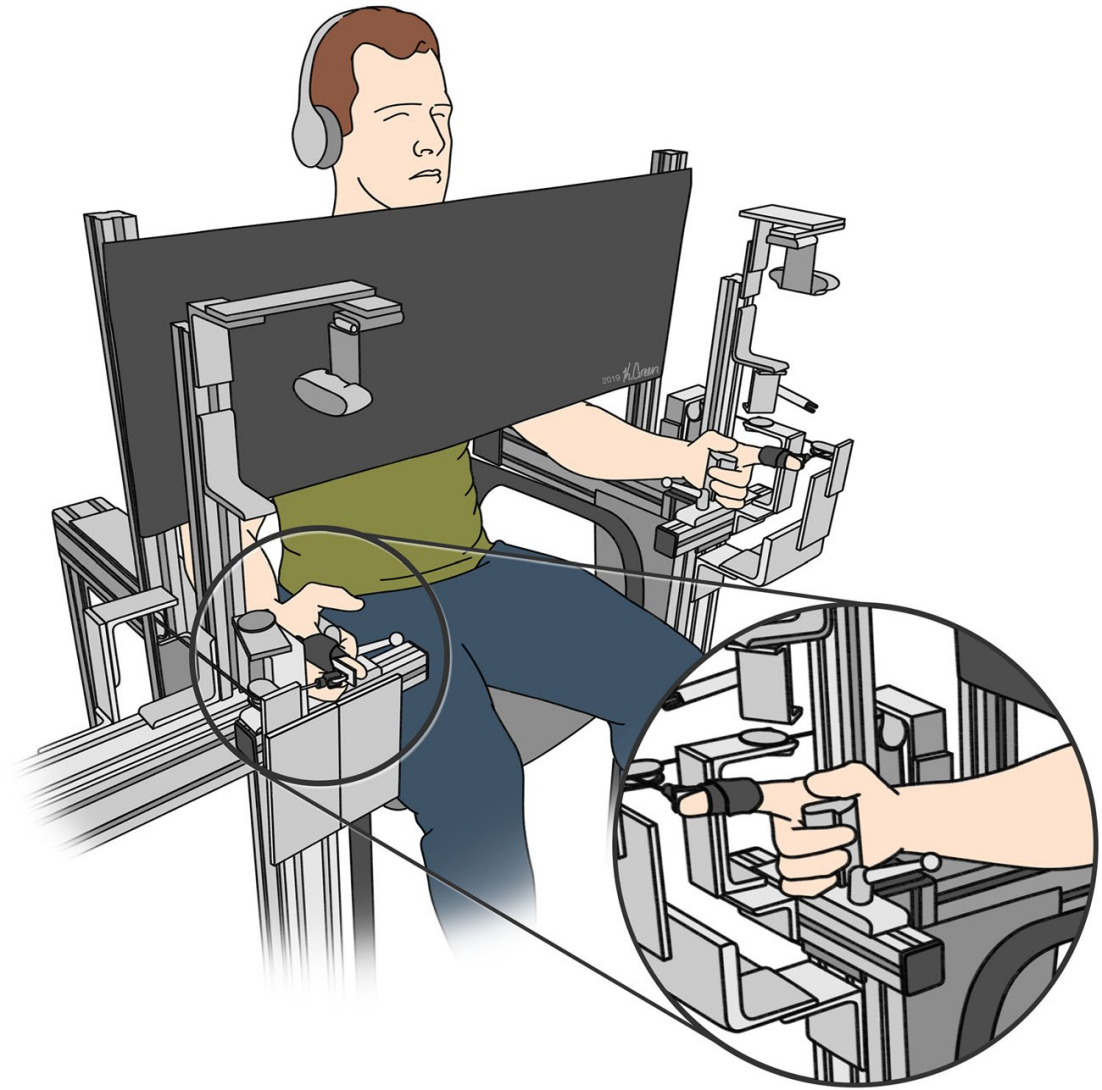
Apparatus. Subjects sat with their forearms resting parallel on two padded beams. The hands were clamped in place such that the palms faced each other. A splint was attached to the dorsal side of each index finger to isolate movement to the MCP joint. A cable ran from the splint through a pulley system, with a platform that allowed for masses to be added to the assembly. The subject’s view of the hands was blocked by a screen. Illustration by Kenzie Green.

Subjects rested both hands and forearms on padded support beams flanking the experimental chair (Fig. 1). Hands were fixed with padded adjustable clamps fastened around each palm. To restrict movement to the metacarpophalangeal (MCP) joint of the index finger, a plastic splint – which spanned the entire dorsal aspect of the finger – was secured to the finger using Velcro straps, thereby preventing movement at the proximal interphalangeal (PIP) and distal interphalangeal (DIP) joints. Between trials, subjects rested their fingers against vertical bars positioned to prevent MCP extension. Two high-definition cameras (Logitech c920, California, USA) secured directly above each hand recorded all hand movements. To apply resistive loads to the finger, we affixed a platform between the cord and potentiometer that supported cylindrical masses. The mass of this assembly was 50 g with no added mass. A curtain suspended approximately six inches in front of the subject obscured vision of the hands in all experiments. Subjects wore earphones playing white noise to eliminate auditory cues.

### Behavioral tasks

#### Joint angle matching

We investigated whether subjects can accurately perceive the position of a given digit, and whether this ability is affected when external forces are applied. In the matching experiment, subjects were instructed to flex their right index finger to one of six predetermined target angles, guided by a visual reference that tracked their movement; maintain the target angle once achieved; then match the resulting position as closely as possible with their left finger. The target angle was cued by a computer monitor, five feet from the subject, which displayed a circle that disappeared when the right finger was within two degrees of the desired angle, as sensed by the voltage signal generated by the linear potentiometer (Honeywell, North Carolina, USA). The mapping between sensor output and angle was calibrated for each subject to account for differences in finger size. Subjects terminated each trial using a foot pedal when they were satisfied with the match between their left and right finger positions. Subjects were not provided feedback on their performance during the experiment.

Additionally, we determined whether actively achieved and passively imposed finger postures were perceived differently. On some experimental blocks, the reference finger was passively set to the target position by the experimenter rather than being actively moved to that position by the subject. Upon passive achievement of the finger position, subjects were instructed to actively maintain that position while attempting to match it with the left index.

Joint angle targets spanned the range from 10 to 60 degrees, separated by 10 degrees, covering most of the typical range of motion of the MCP^15^, broader than that typically evaluated in similar experiments^16,17^. We verified that the distance between target angles exceeded previously measured just noticeable differences for finger angle^16,18,19^. Different matching positions were instructed in pseudo-random order. Trials were grouped in blocks, each with a randomly selected mass (50, 100, 150, and 250 g) imposed on the right (reference) finger. The left (test) finger was subjected to the mass of the experimental apparatus (50 g). The experiment duration was approximately 1.5 hours and consisted of eight blocks (two per weight condition) of 18 trials each (six angles repeated three times each) for a total of 144 trials (36 per weight condition). Subjects were given breaks after every other experimental block.

#### Angle discrimination

While matching experiments are well suited to reveal perceptual biases, they do not provide precise information about sensitivity. To measure joint acuity, we investigated the degree to which subjects could discriminate joint posture across hands, and assessed the degree to which angular acuity was affected by imposed load. Using the experimental apparatus described above, subjects moved the right index to a fixed reference angle (30 degrees) and moved the left to one of eight angular positions spanning the range from 10 to 60 degrees (0, 18, 25, 28, 32, 35, 42, 60), cued in pseudo-random order. Subjects’ movements were guided by the same visual feedback as that described above. Once subjects had actively achieved the reference and target positions on a given trial, they reported which of the two digits was more flexed. Trials were blocked using a similar structure as in the matching experiments. However, only two weights (50 g and 250 g) were imposed on the right (reference) finger, while a constant resistance (50 g), inherent in the pulley apparatus, was imposed on the test finger (as before). Subjects completed two blocks (one per weight condition) of 64 trials each (eight test angles repeated eight times each) for a total of 128 trials.

#### Weight discrimination

The ability to sense the loads applied to and by the hand is essential for dexterous interactions with objects. However, the sensitivity of individual digits to load has not previously been characterized, nor has the influence of finger posture on load perception. To fill this gap, we had subjects perform a weight discrimination task using the experimental apparatus described above. On each trial, subjects moved the left and right index fingers to a target angle of 30 degrees, cued by the previously described visual feedback. The right finger was subjected to one of two reference weights (100 or 250 g) and the left finger was subject to a weight that varied from trial to trial and ranged from 80 to 120% of the reference weight. Reference weights were chosen to span the range of forces required to press a key on a standard keyboard^20^. Trials were blocked into groups of 45 trials, each block with a constant reference weight. Once target positions had been achieved on a given trial, the subject maintained both positions and verbally reported which weight felt heavier.

In separate experiments, we investigated whether sensitivity to changes in load is modulated by finger posture. The setup and task were the same as in the previous weight discrimination study but with a single reference weight (100 g) but with both fingers held at one of two postures (10 or 60 degrees). Subjects completed three blocks of sixty trials (total of 90 trials at each posture).

The experimental setup was designed to minimize cutaneous cues by distributing the loads over large swaths of skin. To verify that tactile cues made only a minor contribution to load discrimination performance, we carried out a control experiment in which subjects fully relaxed both hands while each splint was fixed onto the rig such that the finger was rendered immobile with the MCP held at 0 degrees. Subjects then performed the same discrimination task with weights passively applied to each index finger. Because the MCP was fully supported, any information about load was conveyed via skin deformations delivered through the Velcro straps that held the splint in place.

### Measurement of finger angle

We measured the angle of the MCP using a protractor placed above the hand such that the origin of the protractor was over the center of the knuckle. The subject began each trial with the dorsal aspect of the index finger resting against a metal support, positioned so that the finger was effectively straight relative to the rest of the hand and forearm, and closely aligned with the protractor line indicating zero degrees. During angle matching experiments, we placed four tracking dots along the radial aspect of the finger, straddling each of the MCP, PIP, and DIP joints. These allowed for faithful reconstruction of MCP angle and verification that the PIP and DIP joints were immobile. Using the video from each trial, we identified the final positions of the tracking markers and determined the angle of the MCP from these positions.

### Data analysis

For the angle discrimination experiments, the proportion of trials on which the left index was perceived as more flexed was fit with a logistic function for each participant using the least squares method. For weight discrimination experiments, psychometric functions were similarly fit to the proportion of trials where the weight in the test finger was judged to be “heavier.” Two measures of performance were derived from the psychometric functions. The point of subjective equality (PSE), the test angle or weight at which the subject is equally likely to choose the reference or the test, constitutes a measure of bias. The just noticeable different (JND), corresponding to the change in angle or weight (averaged over increment and decrement) that yields 75% correct discrimination performance, constitutes a measure of sensitivity.

As many of the statistical comparisons did not reject the null hypothesis, we systematically performed power analyses to estimate the sample size – *N*_*sig*_ – required for that effect size to reach statistical significance given 80% power (1-β) and 5% false positive rate (*α*).

## Results

### Perception of joint angle

#### Joint angle matching

First, we measured the perception of joint angle and the degree to which it is influenced by a load imposed on the digit. To this end, subjects were instructed to flex their right index finger until a circle displayed on a monitor disappeared, then match the resulting posture with their left index finger. Resistive weights – ranging from 50 to 250 g – were applied to the reference finger to assess whether load influences perceived posture. Moreover, we compared two conditions: One in which the subject actively moved the reference finger to the reference angle, and one in which the reference finger was passively moved by the experimenter to the reference angle. Matching performance of each individual subject during the active task is shown in Supplemental Figure 1, whereas each individual subject's performance during the passive task is shown in Supplemental Figure 2.

**Figure 2:**
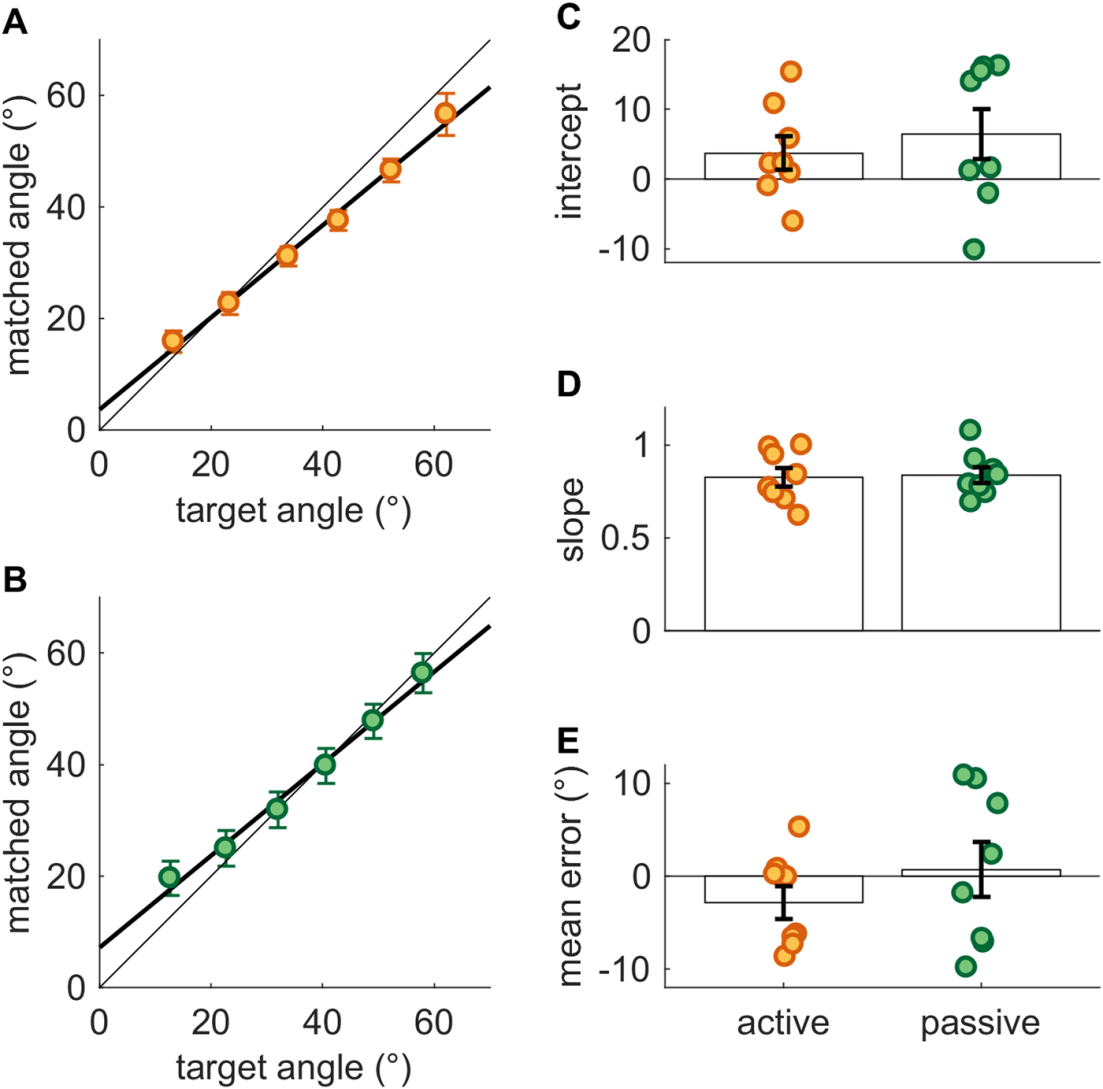
Angle matching. Matching performance under active and passive conditions. The right finger lifted one of four masses (50, 100, 150, or 250 g), and the left finger always lifted 50 g. Angle matching was performed for six target angles (spanning 10 to 60°). Group means in (**A**) active and (**B**) passive angle matching are reported, as are the (**C**) intercept and (**D**) slope of the regression for each subject. (**E**) Mean error, reflecting biases, across all target angles. Error bars denote SEM.

When pooling behavioral data across loads and across active and passive conditions, matched and reference angles were linearly related with a mean slope of 0.83 across subjects (Fig. 2A,B). This slope was slightly, albeit significantly, shallower than unity (one-sample t-test, *t*(15) = 5.37, *p* = 7.8e-05). However, the difference in slopes between the active and passive conditions (0.010) was not significant (two-sample equal-variance t-test, *t*(14) = 0.16, *p* = 0.88). Power analysis reveals that, given 80% power (1-β) and 5% false positive rate (*α*), 5000 subjects distributed across the active and passive conditions would be required for this difference to reach statistical significance (*N*_*sig*_ = 5000).

Matching bias and sensitivity varied widely across subjects in both the active and passive conditions (Fig. 2C,D). However, subjects’ mean signed matching error (−1.1°) was not significantly different from 0° (one-sample t-test, *t*(15) = 0.63, *p* = 0.54, *N*_*sig*_ = 320) (Fig. 2E). The difference in mean signed error between active and passive conditions (3.6°) was also not significant (two-sample equal-variance t-test, *t*(14) = 1.04, *p* = 0.32, *N*_*sig*_ = 118). Matching accuracy – as indexed by the absolute matching error – was also highly variable across subjects, ranging from 4.3° to 11.5°, with a mean of 7.9°.

**Figure 3:**
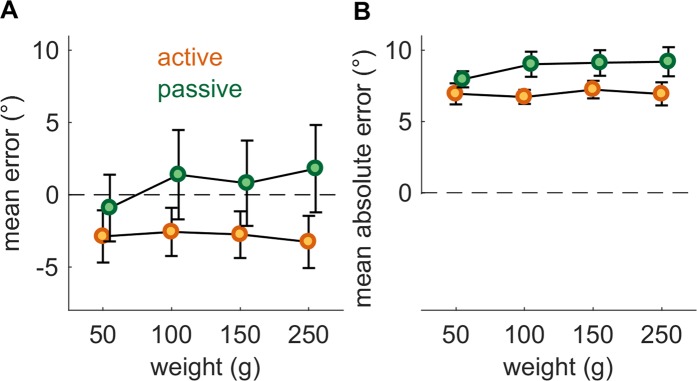
Load dependence of angle matching performance. (**A**) Mean bias (direction of error preserved) vs. imposed load. (**B**) Mean absolute error vs. imposed load. Error bars denote SEM.

We then assessed whether the exertion of force affected the matched posture and found that matching was not affected by load. Indeed, matching bias, as indexed by mean signed error, was not significantly affected by load during the active (repeated measures one-way ANOVA, *F*(3,21) = 0.22, *p* = 0.88) or passive condition (repeated measures one-way ANOVA, *F*(3,21) = 1.47, *p* = 0.25) (Fig. 3A). A slight trend toward positive signed error with heavier weights was observed in the passive condition but even the largest difference–between 50 and 250 g (2.7°) – was non-significant (paired-samples t-test, *t*(7) = 1.65, *p* = 0.14, *N*_*sig*_ = 26) and the difference in signed error between the active and passive conditions at 250 g (5.1°) was also non-significant (two-sample equal-variance t-test, *t*(14) = 1.35, *p* = 0.20, *N*_*sig*_ = 72).

Moreover, matching accuracy, as indexed by absolute matching error, was not significantly affected by load during the active (repeated measures one-way ANOVA, *F*(3,21) = 0.18, *p* = 0.91) or passive condition (repeated measures one-way ANOVA, *F*(3,21) = 0.77, *p* = 0.53) (Fig. 3B). A small difference in the mean absolute error between the active and passive conditions (1.8°) emerges, but this difference is not significant (two-sample equal-variance t-test, *t*(14) = 2.00, *p* = 0.066, *N*_*sig*_ = 34).

In conclusion, subjects can veridically perceive finger posture, this sense is impervious to externally applied loads, and it does not depend substantially on whether posture is achieved actively or imposed on the finger.

### Joint angle discrimination

The angle matching paradigm can reveal perceptual biases but only yields a coarse measure of sensitivity in the absolute matching errors. To more reliably measure sensitivity, we had subjects perform an angular discrimination task. In brief, subjects moved their right index finger to a reference angle and their left index finger to one of a series of test angles (all cued visually in a manner that was uninformative about final posture), then reported which finger was more flexed. Psychometric functions were then fit to the angular discrimination judgments as a function of test angle. From these functions, the point of subjective equality (PSE) – at which judgements of ‘lesser’ or ‘greater’ angle are equally prevalent – and just noticeable difference (JND) – the change in angle that yields 75% correct performance – were derived for each subject. Angle discrimination performance for each individual subject is shown in Supplemental Figure 3.

**Figure 4:**
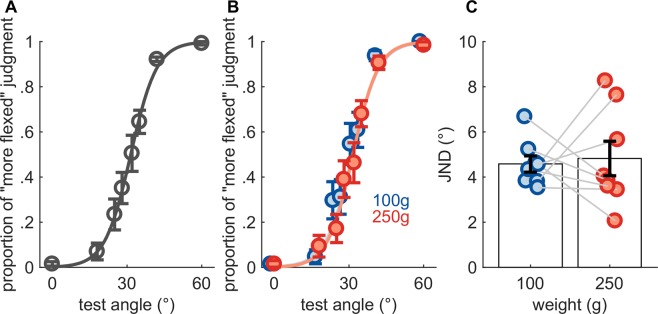
Angle discrimination. (**A**) Mean performance, pooled across load conditions. (**B**) Mean performance split by load condition. (**C**) Just noticeable differences. Faint lines connect data obtained from individual subjects. Error bars denote SEM.

When discrimination judgments were pooled across load conditions, the average PSE (31.2°) was not significantly different from the reference angle (30°) (single sample t-test, t(7) = 0.824, *p* = 0.44, *N*_*sig*_ = 95) (Fig. 4). The typical JND was 4.7° ± 1.0° and ranged from 3.4° to 6.1°. The difference in PSE between loads (0.6°) was not significant (paired-sample t-test, *t*(7) = 0.604, *p* = 0.5649, *N*_*sig*_ = 175), nor was the difference in JND between loads (0.2°) (paired-sample t-test, *t*(7) = 0.257, *p* = 0.8048, *N*_*sig*_ = 956) (Fig. 4C).

In conclusion, subjects exhibited high angular acuity, within half the width of the fingertip, and acuity was insensitive to loads imposed on the finger, consistent with the angle matching results.

### Perception of load

Next, we sought to measure sensitivity to changes in load imposed on the finger. To this end, subjects lifted weights with each index finger and judged which was heavier. On a given block of trials, the weight lifted by the right finger remained constant and served as reference while the weight on the left varied from trial to trial. Each individual subject's weight discrimination performance is shown in Supplemental Figure 4.

**Figure 5:**
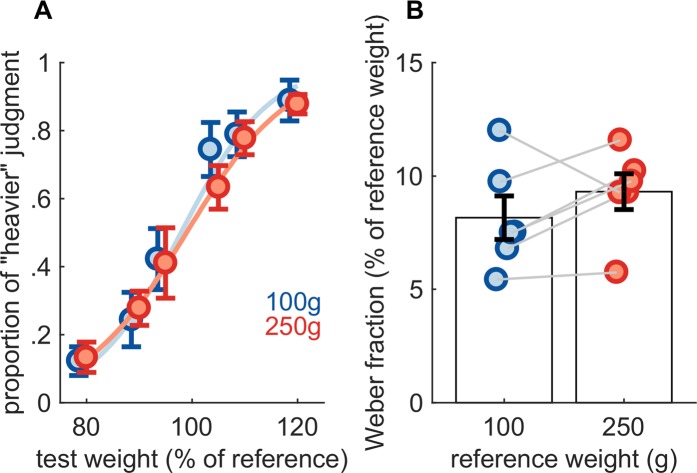
Load discrimination experiment. (**A**) Mean performance. (**B**) Weber fractions. Error bars denote SEM.

When normalized by reference weight then pooled across reference weights, the average PSE (98.4%) was not significantly different from 100% (single sample t-test, *t*(5) = 0.518, *p* = 0.6264, *N*_*sig*_ = 178) (Fig. 5), so there was no systematic bias. Moreover, as might be expected, the JND increased with reference load from 8.2 g for the 100-g reference to 23.3 g for the 250-g reference, yielding a similar Weber fraction (8.2% and 9.3%, respectively) for each. The difference in PSEs between reference weights (0.3%) was not significant (paired-samples t-test, *t*(5) = 0.17, *p* = 0.8757, *N*_*sig*_ = 1741), nor was the difference in Weber fractions (1.1%) (paired-samples t-test, *t*(5) = 1.35, *p* = 0.24, *N*_*sig*_ = 28) (Fig. 5B).

**Figure 6:**
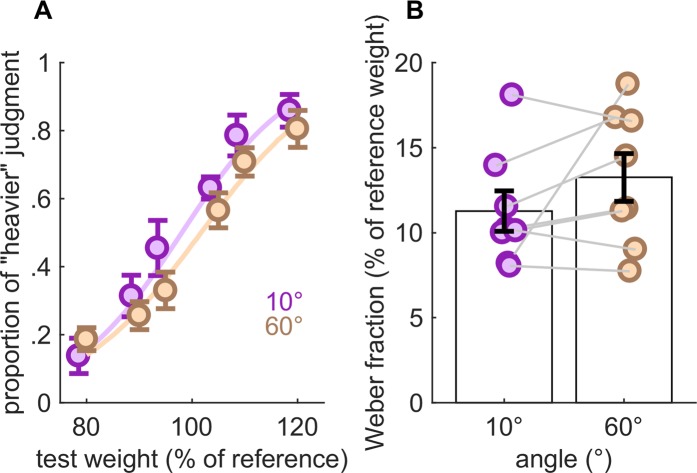
Effect of finger posture on load discrimination. (**A**) Mean performance (**B**) Weber fractions. The reference weight for this experiment was 100 g. Error bars denote SEM.

Next, we examined whether sensitivity to changes in load was affected by joint angle. Each individual subject's weight discrimination performance in the presence of changing joint angles is shown in Supplemental Figure 5. With a 100 g reference weight and the joint flexed at 10 or 60°, the PSE difference across angles (4.1 g) was not significant (paired-samples t-test, *t*(7) = 1.58, *p* = 0.16, *N*_*sig*_ = 28) (Fig. 6). More importantly, JNDs were 11.3 and 13.2 g at 10 and 60°, respectively, yielding a difference between these that was not significant (paired-samples t-test, *t*(7) = 1.46, *p* = 0.19, *N*_*sig*_ = 32). Joint angle has little effect on load discrimination performance, just as load has little effect on joint angle discrimination or matching.

**Figure 7:**
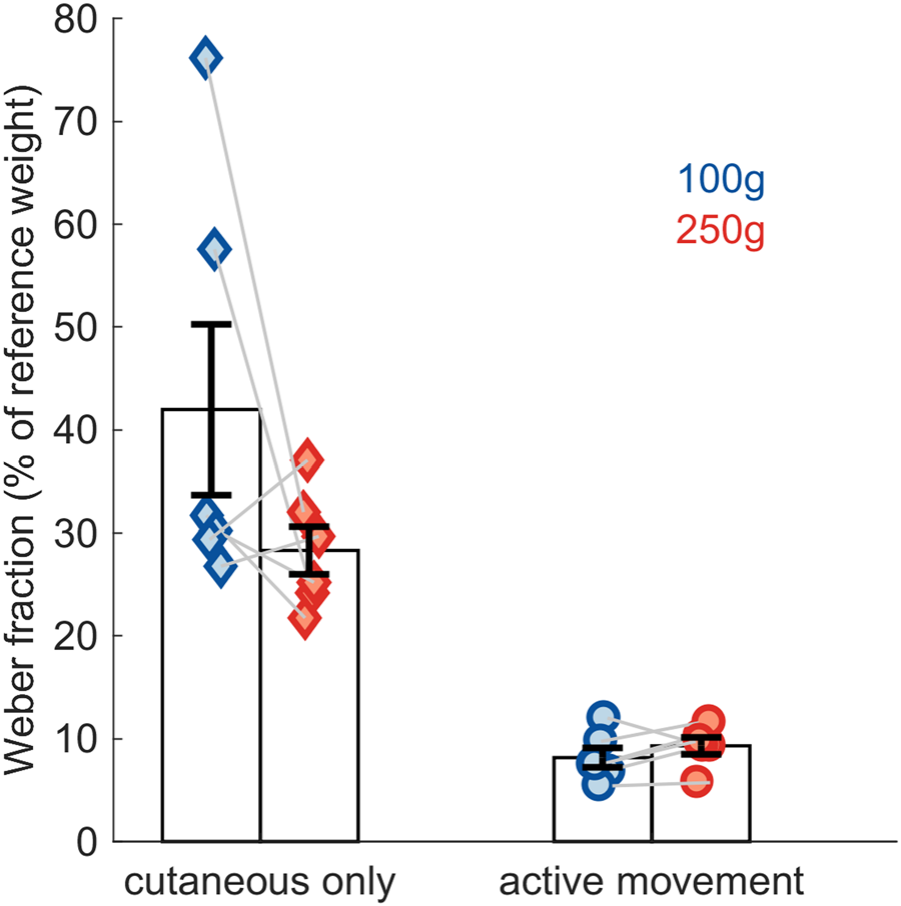
Cutaneous contribution to load discrimination. Performance based on cutaneous cues only compared to when proprioceptive information is available. Error bars denote SEM.

In the load discrimination experiments, the subjects may have relied on tactile cues to perform the task despite our attempts to minimize these. With this in mind, we assessed the extent to which performance on the task could have relied on cutaneous signals. To this end, we applied loads to the subjects’ stationary index finger with the same apparatus, but prevented flexion or extension of the MCP so that all the weight was absorbed by the skin. Under these conditions, when pooling across reference weights, Weber fractions were substantially larger than in the active condition (two-sample equal-variance t-test, *t*(10) = 5.97, *p =* 1.4e-04, Fig. 7). Indeed, JNDs in this control condition were 42.0 g (100-g reference) and 70.8 g (250-g reference), corresponding to Weber fractions of 42.0 and 28.3%, compared to 8.2 and 9.3%, respectively, in the active condition. Thus, cutaneous cues played only a minor role in the subjects’ load discrimination performance in the active condition.

In conclusion, normal load perception at the finger joint is sensitive to small differences (~10%), follows Weber’s law, and is independent of finger posture.

## Discussion

We found that the perceptions of joint angle and that of load – measured at the index finger metacarpo-phalangeal (MCP) joint – were accurate and precise. Furthermore, we did not observe any interactions between posture and load perception despite the fact that muscle spindle afferents – the main source of proprioceptive signals – convey ambiguous information about load and posture. The independence of kinematic and kinetic signals from the hand contrasts with what has been observed with the elbow and shoulder^9,10^ (see below).

### Proprioceptive acuity

Manual interactions with objects require that the hand conform precisely to the object, an ability that entails precise control and tracking of hand postures. Our results suggest that our perception of hand posture is veridical, i.e. does not exhibit any systematic biases, and precise, yielding JNDs down to about 5°. Note that, by our estimate, acuity is poorer than has been previously documented (~1–2° JNDs)^18,19^. This difference could be attributed to the fact that we required subjects to compare simultaneously-presented postures across hands whereas previous studies required subjects to compare sequentially-presented postures on the same finger of the same hand. In one task, the subject has to memorize the position, but tries to reproduce a specific sensory state. In the other, the subject does not have to rely on memory, but has to extract specific sensory information from one hand (joint angle) and transfer it to the other. Our results suggest that the latter results in greater error than the former. Regardless, angle joint perception is highly acute by all accounts.

Manual interactions with objects also require the application of precise forces on objects. We found sensitivity to load to be high, with subjects able to discriminate changes in load on the order of 8 to 9%, within the range of what has been reported previously for load perception at the fingers (9–15%) and higher than for load perception at the proximal limb (14–41%)^21^. These accounts suggest that load perception is highly acute with the fingers, more so than with the proximal limb.

### Tactile and proprioceptive contributions to the sense of force

We are able to exert a precise amount of force on objects, just enough to pick them up without dropping them^22^. This ability is thought to rely in large part on tactile cues, as evidenced by the much higher forces exerted on objects when cutaneous input is removed through digital anesthesia^23^. However, proprioceptors also offer a source of information regarding the amount of force being imposed by the hand. In the present study, we wished to measure the sensitivity to load conveyed through the proprioceptive sense, so we deliberately minimized cutaneous cues by distributing the load over a wide swath of skin. That Weber fractions quadrupled when only tactile cues were available demonstrates that we succeeded. The present results demonstrate that precise force information is also available from proprioception. Note that precise load perception was achieved despite the fact that the MCP joint moved very little, as has been shown with the thumb and elbow^24,25^.

### Active and passive movements

Spindle afferents, the primary source of proprioceptive signals^3^, are modulated by top-down motor signals. One would expect the perception of actively generated postures to be different from that of passively imposed ones, as both are associated with qualitatively different top-down inputs. Surprisingly, the perception of finger posture was similar under active and passive conditions, in contrast to the proximal limb, where postural perception is influenced by whether those postures were achieved actively or passively^12^. One possible explanation for this discrepancy is that hand proprioception relies more heavily on non-spindle channels of postural information, for example cutaneous afferents^18,26–28^.

While our results – that postural perception of the fingers is similar under active and passive conditions – replicate previous findings^17^, motor intent can influence perceived finger posture when those motor commands are entirely prohibited from generating movement, for example in the case of an isometric contraction or when the hand is paralyzed^17,29–31^. Therefore, a bias in finger postural perception likely requires a stark mismatch between expected and achieved finger posture, rather than the mere presence or absence of intent that we tested.

### Independent load and posture perception

We find that the perception of a load imposed on the finger is robust to changes in joint angle. The lack of an influence of posture on perceived load, also observed at the ankle joint^32^, is perhaps unsurprising, as the most likely load-signaling proprioceptor is the Golgi tendon organ (GTO), which tracks muscle tension^33,34^ and does not receive direct efferent input. However, people generally estimate the weight of objects with oscillatory movements and their perceptual acuity drops when they are no longer permitted to make such movements^24^, suggesting that signals from traditionally kinematic receptors such as muscle spindles may also participate in load perception (though these oscillations may simply allow for repeated sampling). Nonetheless, our findings suggest that, to the extent such spindle signals might be used to generate a load percept, any ambiguity arising from multiplexed kinematic and muscle effort signals in those spindles does not contaminate load perception.

We also find, more surprisingly, that the acuity of the finger postural sense is robust to changes in load and that postural perception is the same whether the posture was achieved actively or imposed on the finger (but maintained actively). These findings are consistent with other work that attempts to dissociate the senses of load and position^17,35,36^ and imply a synthesis of multiple channels of information in proprioception, as is the case with touch^37,38^. Indeed, while spindle afferents constitute the dominant source of proprioceptive signals, they respond more readily during active than passive movement^39^ and their spiking is dependent on the load supported (and hence effort exerted) by the limb in a manner not entirely predicted by alpha-gamma co-activation^40^. As such, they provide an ambiguous signal of limb position if taken in isolation. These ambiguities could, in principle, be resolved by (1) integrating information from GTO afferents^41^, presumably to estimate and then correct for the degree to which the spindle response can be attributed to force generation^17^, (2) relying on skin stretch^26–28^ or other cutaneous signals^18^, which offer poor postural acuity on their own^42,43^ but are uncontaminated by efferent signals, and (3) using a forward model^22,44^, which permits not only rapid behavioral adaptation to violations of sensory expectations, but could also assist in the disambiguation of hand postural information from the responses of spindle afferents. The synthesis of multiple channels of information to support kinesthesia is supported by muscle afferent recordings, which show that joint angle and angular velocity are better decoded from the responses of multiple populations of proprioceptive afferents than they are from the responses of any one population^41,45^.

### Proprioception of the proximal and distal limb

We find that load perception is more acute for the fingers than it is for the proximal arm (i.e., shoulder and elbow)^21^. Moreover, there does not appear to be an effect of motor intent *per se* on perceived finger posture, unlike what is seen in the proximal arm^12^. There is also a lack of interaction between load and joint posture signals from the finger, even when they do seem to interact for the lower limb and proximal arm^5,9,10^. At first glance, this is surprising: finger proprioception arises in part from receptors in the extrinsic muscles of the hand, whose insertions are located as far proximal as the distal humerus. Given how far the hand musculature extends, hand proprioception should not be qualitatively different from its proximal counterpart.

However, factors beyond muscular anatomy could support perceptual invariance in hand and finger, but not proximal limb, proprioception. First, evidence suggests that cutaneous cues such as skin stretch seem to influence the perceived posture of the fingers more than they do that of the elbow or knee^26,27,46,47^. Such information could, in principle, play a role in disambiguating postural information from kinetic and descending motor control signals in the proprioceptive periphery. Additionally, the different functional roles of the proximal limb and fingers likely entail different sensory requirements. Indeed, the proximal limb is tasked with hand transport in 3D space, whereas the hand is tasked with conforming to and manipulating objects of various shapes and sizes. The hand mediates stereognosis – the recognition of the three-dimensional shape of objects – which requires a precise, accurate, and robust representation of hand posture^48,49^.

## Conclusions

We conclude that the perception of finger posture and the perception of loads imposed on the finger are highly precise, as might expected given the function of the hand. Furthermore, we find that posture perception is independent of loads imposed on the finger, and load perception is independent of the posture of the finger, despite the fact that the principal source of proprioceptive signals – muscle spindles – multiplex these two types of information. These results are consistent with a model according to which both postural and load perception involve the synthesis of multiple sources of sensory information, and point to the particular importance of such synthesis for proprioception of the fingers and hand.

## Supplementary information

Supplementary figures
