# Supplementary material for: Finger Posture and Finger Load are Perceived Independently
Source: Sci Rep. 2019 Oct 21;9:15031. doi: 10.1038/s41598-019-51131-x (PMC6803715; doi:10.1038/s41598-019-51131-x)
Supplement: Supplementary file 1 — Supplementary figures [file 41598_2019_51131_MOESM1_ESM.docx]

Finger posture and finger load are perceived independently

Brendan Prendergast^1^, Jack Brooks^1^, James M. Goodman^2^, Maria Boyarinova^1^, Jeremy E. Winberry^1^, & Sliman J. Bensmaia^1,2^

^1^Department of Organismal Biology and Anatomy, University of Chicago, Chicago, IL

^2^Committee on Computational Neuroscience, University of Chicago, Chicago, IL

**Supplementary Information**


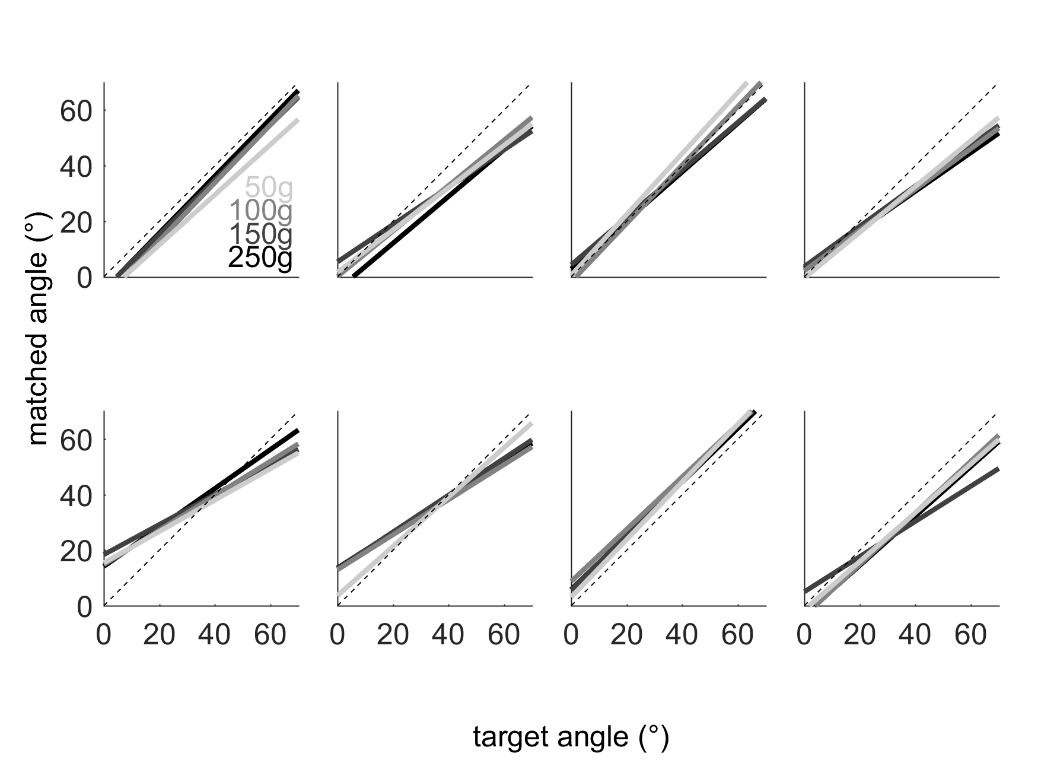


Supplemental Figure 1: Angle matching performance in the active movement condition. Both fingers were loaded with 50, 100, 150, or 250 g masses (light to dark lines, dashed line is unity). Each panel corresponds to a single subject.


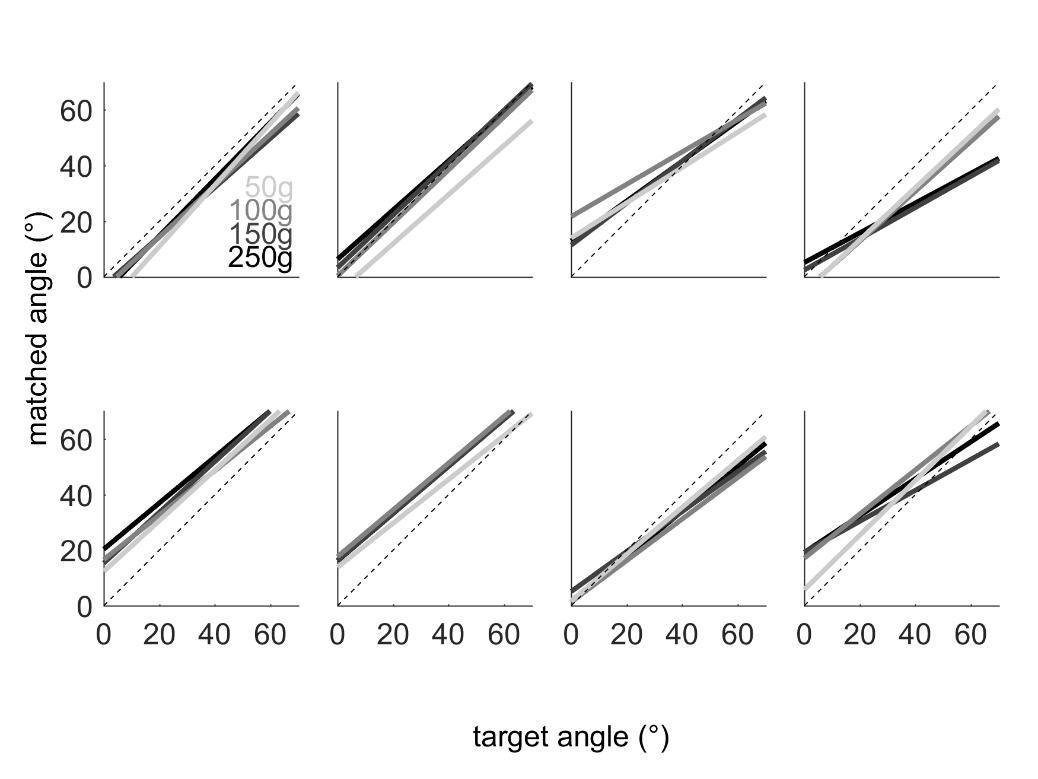


Supplemental Figure 2: Angle matching performance in the passive movement condition. Both fingers were loaded with 50, 100, 150, or 250 g masses (light to dark lines, dashed line is unity). Each panel corresponds to a single subject.


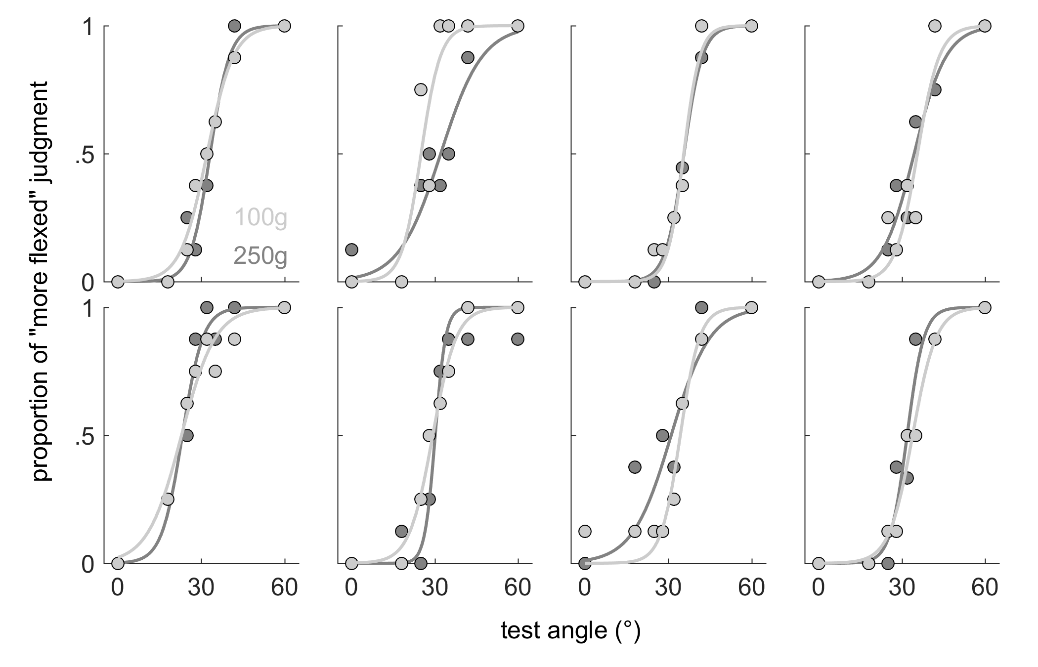


Supplemental Figure 3: Angle discrimination performance. The right finger always moved to 30 degrees, and the left finger one of eight test angles. The right finger was loaded with 100 or 250g, whereas the left finger actively moved against a 50g load. Each panel corresponds to a single subject.


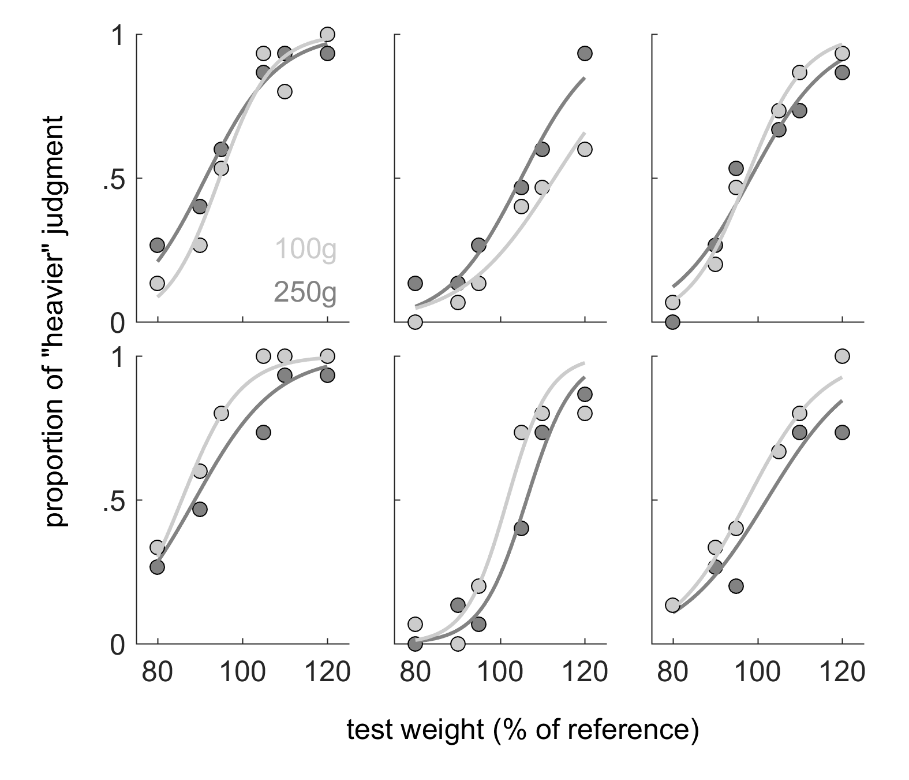


Supplemental Figure 4: Weight discrimination performance. The right finger was loaded with a 100g or 250g reference weight, whereas the left finger was loaded with one of a range of test weights. Each panel corresponds to a single subject.


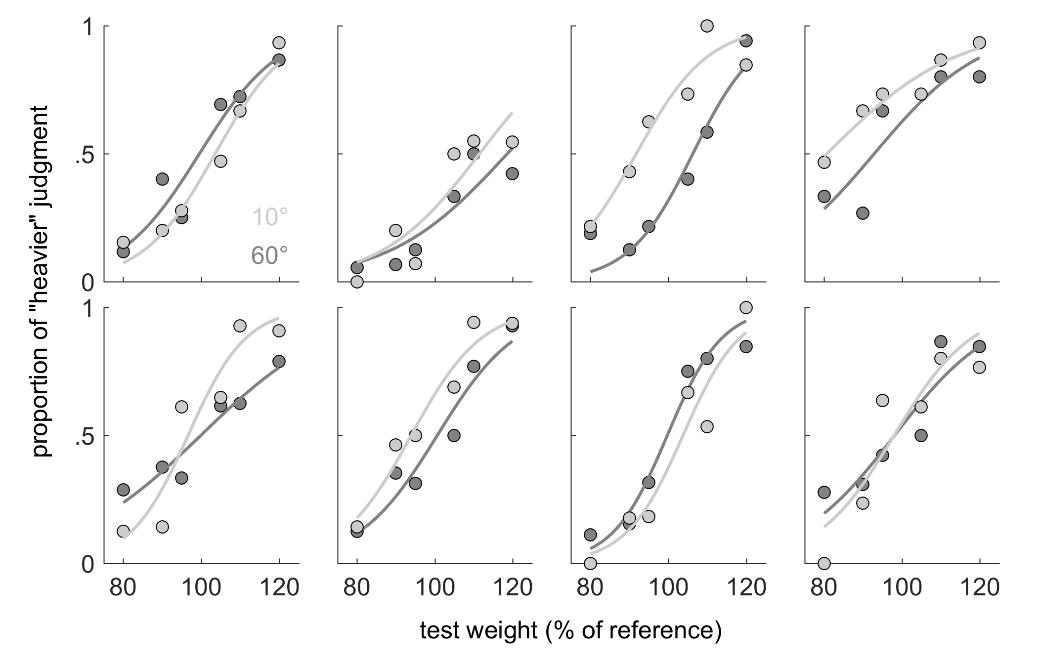


Supplemental Figure 5: Weight discrimination performance at two test angles. Both fingers were moved to 10 or 60°. The right finger was always loaded with a 100g reference weight, whereas the left finger was loaded with one of a range of test weights. Each panel corresponds to a single subject.
